# Supplementary material for: Design and performance characteristics of the Elecsys anti-SARS-CoV-2 S assay
Source: Front Immunol. 2022 Dec 2;13:1002576. doi: 10.3389/fimmu.2022.1002576 (PMC9756759; doi:10.3389/fimmu.2022.1002576)

***Supplementary material***

**Supplementary Table S1. Cross-reactivity of the ACOV2S assay in samples from patients with non-respiratory infections.**

| **Indication** | | | **Samples (n)** | **Reactive in Elecsys Anti-SARS-CoV-2 (n)** | **Specificity** |
| --- | --- | --- | --- | --- | --- |
| ***Other infectious diseases:*** | | | | | |
| Adenovirus | | | 25 | 0 | 100.0% |
| Borrellia | | | 6 | 0 | 100.0% |
| Candida albicans | | | 13 | 0 | 100.0% |
| Chlamydia trachomatis | | | 12 | 0 | 100.0% |
| CMV acute (IgM+ IgG+) | | | 86 | 0 | 100.0% |
| E.coli (anti-E.coli reactive) | | | 10 | 0 | 100.0% |
| EBV acute (IgM+ VCA IgG+) | | | 106 | 0 | 100.0% |
| Gonorrhea (Tripper) | | | 5 | 0 | 100.0% |
| HAV acute (IgM+) | | | 10 | 0 | 100.0% |
| HAV late (IgG+) | | | 15 | 0 | 100.0% |
| HAV vaccinees | | | 15 | 0 | 100.0% |
| HBV acute | | | 12 | 0 | 100.0% |
| HBV chronic | | | 12 | 0 | 100.0% |
| HBV vaccinees | | | 15 | 0 | 100.0% |
| HCV | | | 50 | 0 | 100.0% |
| HEV | | | 12 | 0 | 100.0% |
| HIV infection | | | 10 | 0 | 100.0% |
| HSV acute (IgM+) | | | 24 | 0 | 100.0% |
| HTLV | | | 6 | 0 | 100.0% |
| Legionella (IgGAM+) | | | 7 | 0 | 100.0% |
| Listeria | | | 6 | 0 | 100.0% |
| Measles | | | 10 | 0 | 100.0% |
| Mumps | | | 14 | 0 | 100.0% |
| Parvovirus B19 | | | 30 | 0 | 100.0% |
| Plasmodium falciparum (Malaria) | | | 8 | 0 | 100.0% |
| Rubella acute (IgM+, IgG+) | | | 12 | 0 | 100.0% |
| Toxoplasma gondii (IgM+, IgG+) | | | 8 | 0 | 100.0% |
| Treponema pallidum (Syphilis) | | | 62 | 0 | 100.0% |
| VZV (Varicella zoster) | | | 30 | 0 | 100.0% |
| ***Auto-immune diseases:*** | | | | | |
| AMA (anti-mitochondrial antibodies) | | | 30 | 0 | 100.0% |
| ANA (anti-nuclear antibodies) | | | 17 | 0 | 100.0% |
| Hemophiliacs | | | 15 | 0 | 100.0% |
| RA (rheumatoid arthritis) | | | 10 | 0 | 100.0% |
| SLE (systemic lupus erythematosus) | | | 10 | 0 | 100.0% |
| ***Hepatic diseases:*** | | | | | |
| Alcohol induced hepatitis /cirrhosis | | | 13 | 0 | 100.0% |
| Drug induced hepatitis /cirrhosis | | | 10 | 0 | 100.0% |
| Fatty liver | | | 10 | 0 | 100.0% |
| Liver cancer | | | 10 | 0 | 100.0% |
| Non viral liver disease | | | 15 | 0 | 100.0% |
|  |  |  |  |  |  |
| **total** |  |  | **1468** | **0** | **100.0%** |

**Supplementary Table 2. Potential drug interferences in the ACOV2S assay.** Commercially available human plasma samples taken from convalescent individuals following a native SARS-CoV-2 infection in 2020 were spiked with drugs of interest and ACOV2S antibody recovery was calculated at two levels of ACOV2S titer: low (~0.5–4 U/mL, yellow) and high (~150–270 U/mL, blue). Drugs tested by initial application of their 3x daily dose and compared to untreated reference. If required, drugs were solubilized and the reference was supplemented with the equal volume of applied solvent only. Deviation of results of more than 10% at 3x daily dose was further investigated by application of 1x daily dose (regorafenib, ritonavir). Results were generated with commercially available samples (human plasma) from convalescent individuals following native infection in 2020.

|  |  | **Low ACOV2S titer** | **High ACOV2S titer** |  |
| --- | --- | --- | --- | --- |
|  |  | **Recovery (%)** | **Recovery (%)** |  |
| **Drug** | **Drug conc. (mg/L)** |  |  |  |
| **drugs with postulated potential to interrupt the RBD-ACE2 interface** | | | | |
| Risperidon | 0.03 | 92.9 | 90.7 |  |
| Sitagliptin | 0.12 | 92.7 | 90.8 |  |
| Baricitinib | 4.8 | 99.1 | 91.1 |  |
| Silodosin | 9.6 | 94.8 | 93.0 |  |
| Ebastin | 24 | 94.7 | 94.1 |  |
| Indacaterolmaleat | 0.36 | 94.9 | 99.0 |  |
| Regorafenib (3x daily dose) | 0.192 | 89.8 | 90.8 |  |
| Regorafenib (1x daily dose) | 0.064 | 97.5 | n.a |  |
| Omalizumab | 0.18 | 99.4 | 98.0 |  |
| **Special drugs used in COVID-19 treatment** | | | | |
| Zanamivir | 0.006 | 100.1 | 99.6 |  |
| Oseltamivir | 0.090 | 99.9 | 100.0 |  |
| Ceftriaxone | 2.40 | 98.0 | 96.7 |  |
| Levofloxacin | 0.300 | 101.2 | 100.3 |  |
| Meropenem | 3.60 | 101.1 | 100.0 |  |
| Ribavirin | 0.720 | 103.8 | 100.3 |  |
| Azithromycin | 0.300 | 104.2 | 99.4 |  |
| Arbidol | 0.120 | 100.6 | 97.3 |  |
| Lopinavir | 0.720 | 98.2 | 95.3 |  |
| α-interferon 2b | 3000 IE/mL | 100.0 | 99.1 |  |
| Peramivir | 0.360 | 98.4 | 98.8 |  |
| Tobramycin | 0.360 | 102.1 | 101.0 |  |
| Histamine Dihydrochl. | 0.0006 | 99.5 | 101.1 |  |
| Tocilizumab | 0.384 | 99.2 | 100.4 |  |
| α-interferon 2a | 43200 IE/mL | 102.2 | 100.9 |  |
| Hydroxychloroquinsulfat C1 | 0.240 | 96.0 | 99.5 |  |
| Remdesivir | 0.120 | 98.6 | 99.5 |  |
| Ritonavir (3x daily dose) | 0.480 | 74.5 | 68.4 |  |
| Ritonavir (1x daily dose) | 0.160 | 95.5 | 92.4 |  |
| **Common drugs** | | | | |
| Acetylcysteine | 150 | 100.8 | 100.0 |  |
| Acetylsalicylic acid | 30 | 102.6 | 102.7 |  |
| Ampicillin | 75 | 103.5 | 100.6 |  |
| Ascorbic acid | 52.5 | 102.8 | 101.3 |  |
| Cefoxitin | 750 | 101.9 | 101.3 |  |
| Doxycycline | 18 | 103.1 | 105.7 |  |
| Heparin | 3300 IU/L | 103.3 | 101.2 |  |
| Levodopa | 7.5 | 103.4 | 101.0 |  |
| Methyldopa | 22.5 | 103.2 | 100.3 |  |
| Metronidazole | 123 | 102.5 | 100.8 |  |
| Rifampicin | 48 | 103.3 | 100.0 |  |
| Acetaminophen | 156 | 100.4 | 98.6 |  |
| Cyclosporine | 1.8 | 100.9 | 91.8 |  |
| Ibuprofen | 219 | 100.3 | 92.9 |  |
| Theophylline | 60 | 100.3 | 90.7 |  |
| Phenylbutazone | 321 | 104.5 | 93.9 |  |

**Supplementary Figure S1. Correlation of the ACOV2S assay with results from the cPass neutralization assay.** ACOV2S titers and percentage inhibition from the cPass assay from longitudinal samples from individual donors


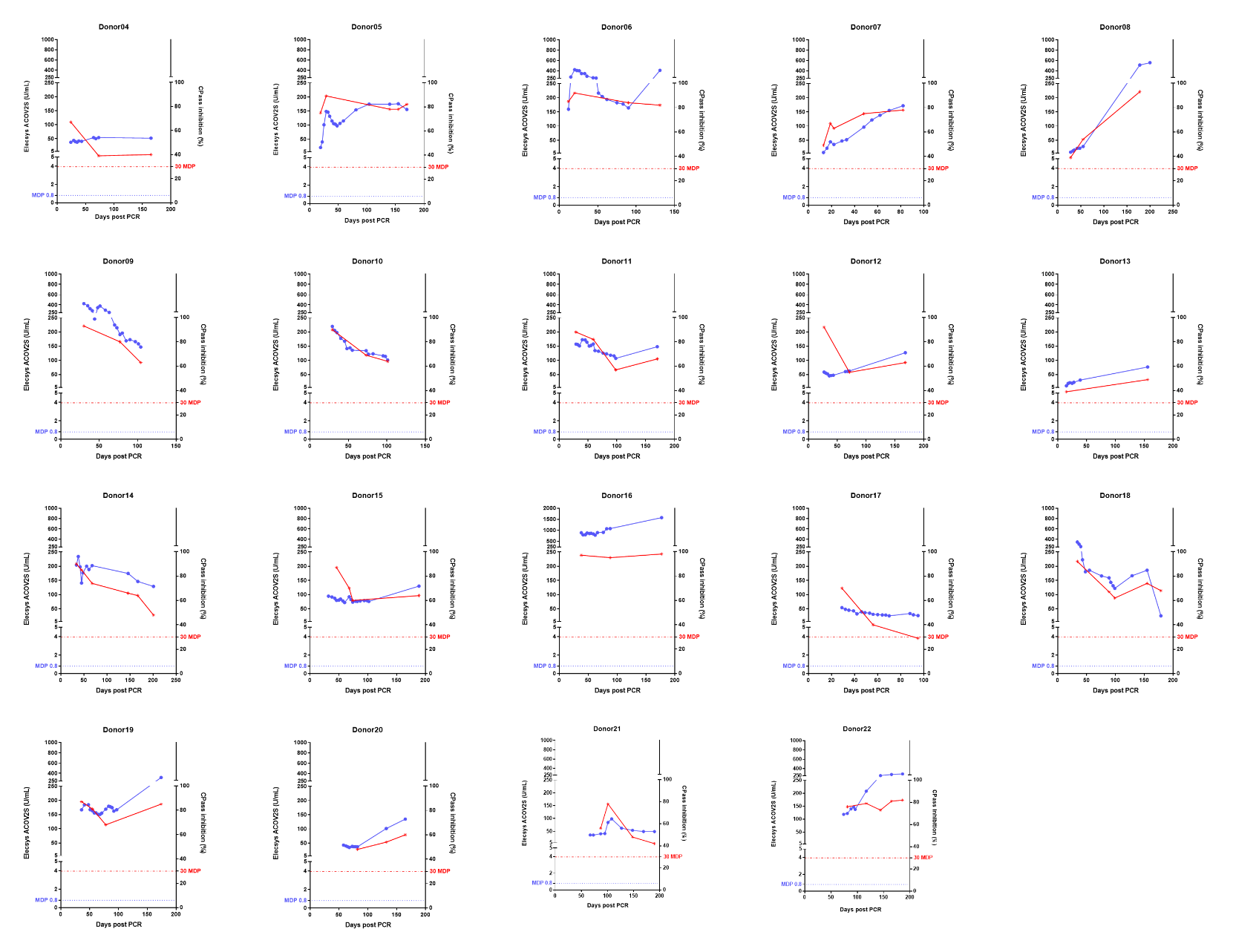


**Supplementary Figure S2. Correlation of the Genscript cPass and Elecsys ACE2-RBD neutralization assays.** (A) Method comparison with cutoffs marked (cPass 30%, ACE2-RBD, 25%), (B) residual plot, and (C) ROC curve. Red circles indicate samples from patients with severe COVID-19 (hospitalized) and blue triangles indicate samples from patients with mild COVID-19 (not hospitalized).

**
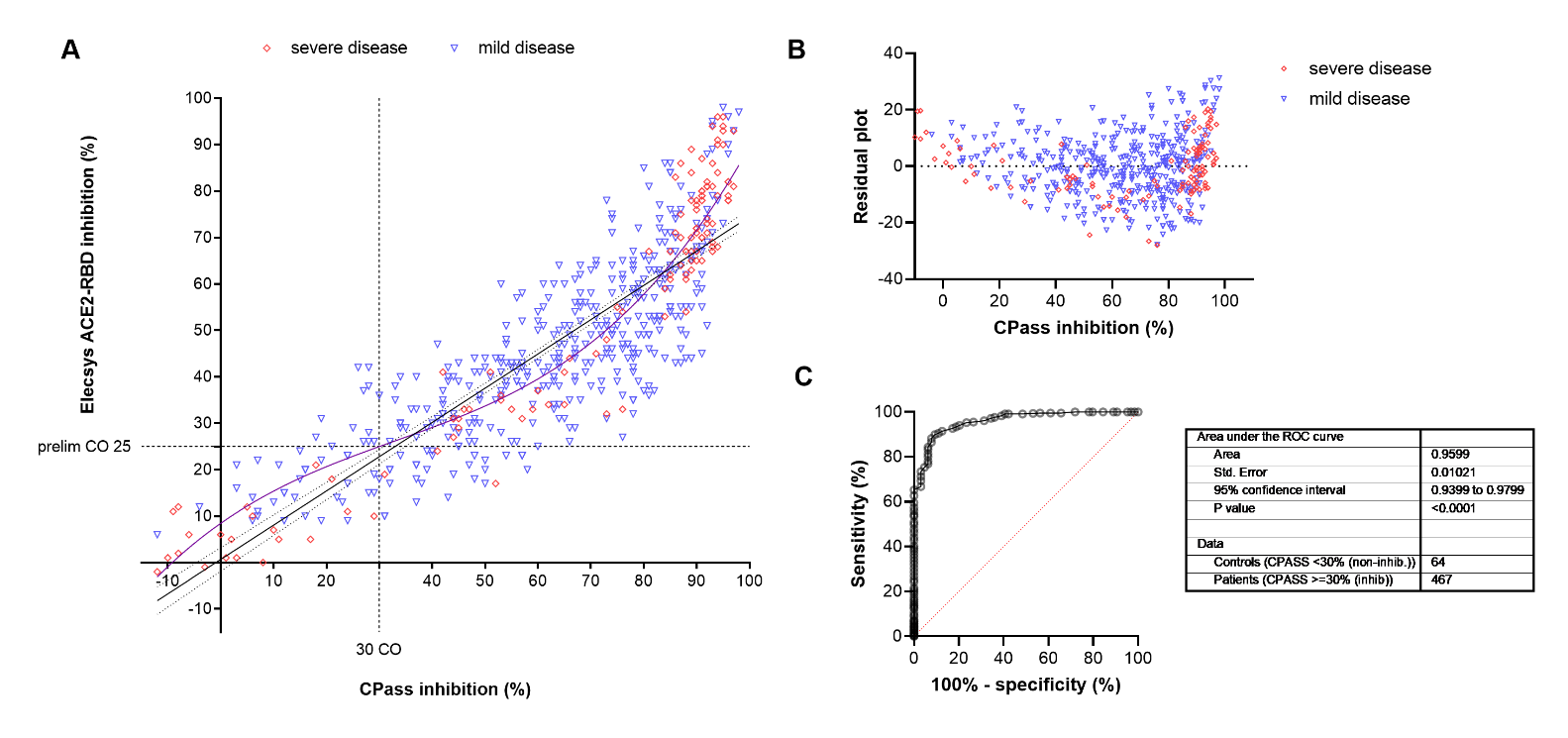
**


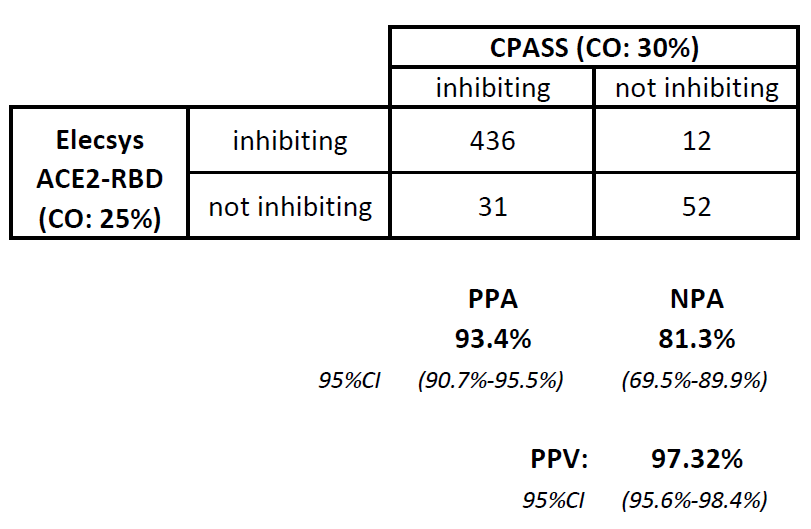
**D**.

**Supplementary Figure S3. Correlation of the Elecsys ACOV2S assay and Elecsys ACE2-RBS neutralization assay.** (A) Method comparison with cutoffs marked (ACOV2S 0.8 U/mL and 15 U/mL, ACE2-RBD, 25%), (B) residual plot, (C) ROC curve, and (D) qualitative agreement between the ACOV2S and ACE2-RBS assays. Red circles indicate samples from patients with severe COVID-19 (hospitalized) and blue triangles indicate samples from patients with mild COVID-19 (not hospitalized).


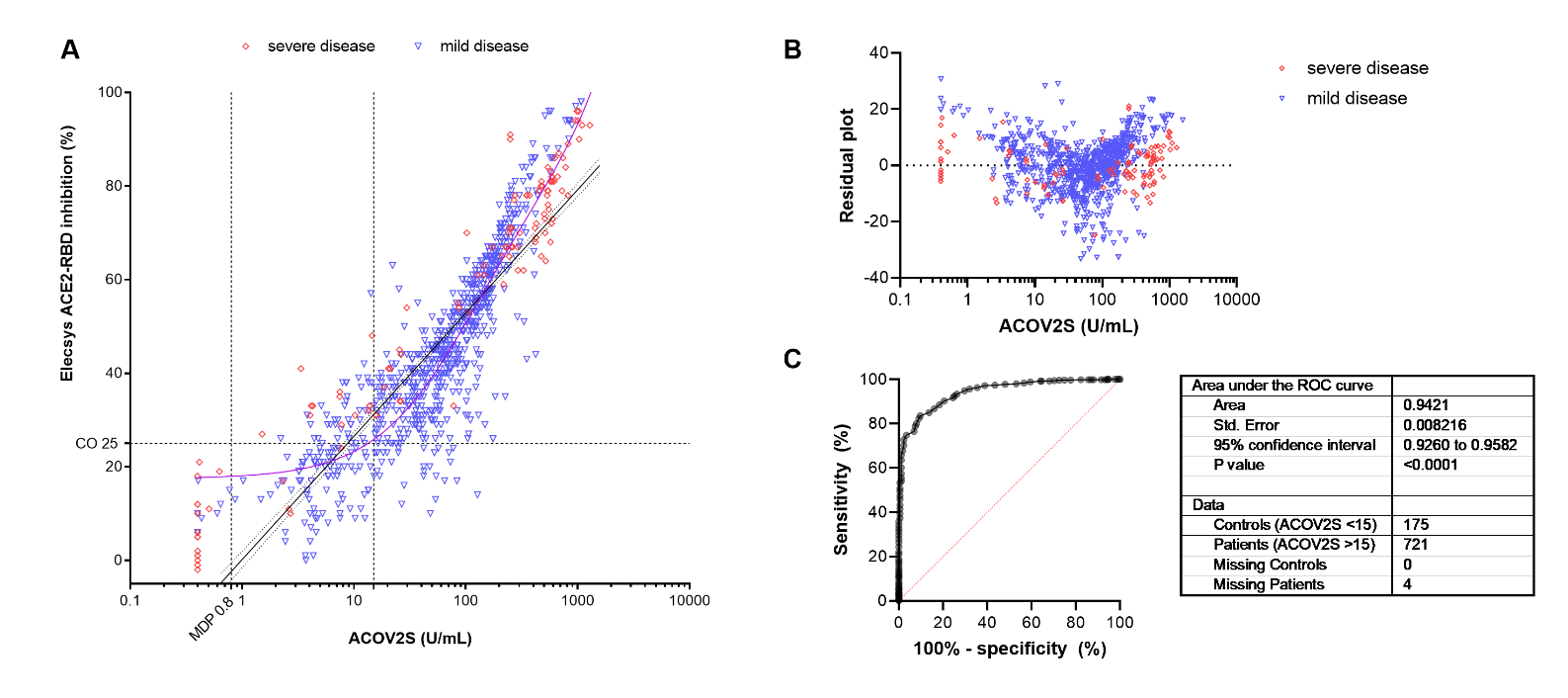


**D)**


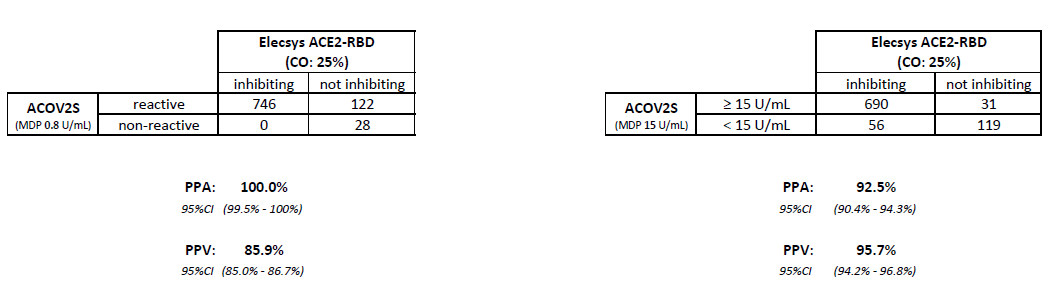

Supplement: Supplementary file 1 [file DataSheet_1.docx]
